# The standardization of a new Explicit Pornographic Picture Set (EPPS)

**DOI:** 10.3758/s13428-024-02418-z

**Published:** 2024-05-01

**Authors:** Sabine Prantner, Cristina Giménez-García, Alejandro Espino-Payá, Miguel A. Escrig, Elisabeth Ruiz-Padial, Rafael Ballester-Arnal, M. Carmen Pastor

**Affiliations:** 1 Departamento de Psicología Básica, Clínica y Psicobiología, Facultad de Ciencias de la Salud, Universitat Jaume I, Castelló de la Plana, Spain; 2 Institute for Biomagnetism and Biosignalanalysis, University of Münster, Münster, Germany; 3 Departamento de Psicología, Facultad de Ciencias de la Salud, Universidad Europea de Valencia, Valencia, Spain; 4 Departamento de Psicología, Universidad de Jaén, Jaén, Spain

**Keywords:** Affective ratings, Picture set, Pornography, Visual sexual stimuli, Sexual attraction

## Abstract

Pictures with affective content have been extensively used in scientific studies of emotion and sexuality. However, only a few standardized picture sets have been developed that offer explicit images, with most lacking pornographic pictures depicting diverse sexual practices. This study aimed to fill this gap through developing a standardized affective set of diverse pornographic pictures (masturbation, oral sex, vaginal sex, anal sex, group sex, paraphilia) of same-sex and opposite-sex content, offering dimensional affective ratings of valence, arousal, and dominance, as well as co-elicited discrete emotions (disgust, moral and ethical acceptance). In total, 192 pornographic pictures acquired from online pornography platforms and 24 control IAPS images have been rated by 319 participants (*M*_age_ = 22.66, *SD*_age_ = 4.66) with self-reported same- and opposite-sex sexual attraction. Stimuli were representative of the entire affective space, including positively and negatively perceived pictures. Participants showed differential affective perception of pornographic pictures according to gender and sexual attraction. Differences in affective ratings related to participants’ gender and sexual attraction, as well as stimuli content (depicted sexual practices and sexes). From the stimuli set, researchers can select explicit pornographic pictures based on the obtained affective ratings and technical parameters (i.e., pixel size, luminosity, color space, contrast, chromatic complexity, spatial frequency, entropy). The stimuli set may be considered a valid tool of diverse explicit pornographic pictures covering the affective space, in particular, for women and men with same- and opposite-sex sexual attraction. This new explicit pornographic picture set (EPPS) is available to the scientific community for non-commercial use.

## Introduction

The elicitation of emotions by visual stimuli is considered an important and very useful tool in psychological, neuroscientific, and clinical research, whereby these stimuli must be carefully selected depending on the design and research question (Gerrards-Hesse et al., 1994; Mauss & Robinson, 2009; Scherer, 2005).

Since the late 1990s, a handful of pictorial stimuli have been made available to the scientific community, which have been rated on affective rating scales by participants under certain standardized conditions (e.g., International Affective Picture System (IAPS) by Lang et al., 1999). However, few of these standardized picture sets provided stimuli with highly sexually explicit depictions, although with the advent of the Internet, pornographic content with an infinite number of different sexual scenarios has spread rapidly (Döring, 2009). The Internet has become a distribution channel for various forms of pornography, which is now easily accessible and usually free of charge, increasing its consumption worldwide and prompting problematic behaviors, such as lower sexual and relationship satisfaction, compulsive sexual behaviors (e.g., addiction like pornography consumption), and increased diverse sexual risk behaviors (Ballester-Arnal et al., 2017, 2022; Baranowski et al., 2019; Bártová et al., 2021; Daneback et al., 2009; Giménez-García et al., 2021; Hald, 2006; Martyniuk et al., 2016). The utilization of visual sexual stimuli has the potential to advance our comprehension of psychophysiological mechanisms underlying human sexual behavior and related disorders. Such research can contribute to shedding light on the phenomenology and neurobiological foundations of these conditions (Kowalewska et al., 2018).

The increasing popularity of pornographic material, and growing exposure to it, also made experimental research in this area relevant. The perception of highly explicit content is well investigable with visual sexual stimuli. In clinical and therapeutic settings, sexual stimuli can be used as part of treatment, and in forensic clinical settings, sexual stimuli may be used to evoke sexual response to provide an objective assessment of atypical sexual interests, for example, in nonconsenting sexual activities (Katz et al., 2023). Thus, sexual stimuli are not only useful in studying the effects of pornography consumption or sexual interests, but also in various basic and clinical studies related to human sexuality – whether in the field of functional neuroimaging, psychophysiology, or subjective assessment of perceived sexual arousal and the sexual response cycle – and can themselves also be used as control stimuli to elicit highly arousing affect in studies unrelated to sexuality research.

Nevertheless, efforts to provide sexual stimuli for other researchers have been rather limited (Cui et al., 2021; Daoultzis & Kordoutis, 2022; Jacob et al., 2011; Lang et al., 1999; Wierzba et al., 2015). Standardized visual sexual stimuli are scarce, and the few stimuli that are available are often not very explicit and do not adequately represent sexual diversity. Many of these stimuli are increasingly outdated; many of the images were collected several decades ago and may therefore be rather unsuitable for experimental research today, partly due to habituation but mainly due to changes in the way sexual content is represented (Jacob et al., 2011). In addition, only a limited amount of non-heteronormative content is available, such as images of same-sex couples in sexual interactions (e.g., NAPS ERO by Wierzba et al., 2015). This does not help to reduce bias in experimental studies that focus predominantly on men and heteronormative representations of sexuality (van’t Hof & Cera, 2021; Georgiadis & Kringelbach, 2012; Poeppl et al., 2016; Stoléru et al., 2012; Ziogas et al., 2020).

The perception and processing of sexual stimuli is related to the picture content and the observer's sexual orientation and gender identity (Wierzba, et al., 2015). Apart from that, studies using visual sexual stimuli to examine sexuality in the context of gender differences and sexual orientation are also difficult to compare, as the pictures used were often not properly controlled for a priori in this sense (Rupp & Wallen, 2009). So, it remains uncertain whether the observed differences represent different ways of arousal processing or merely different levels of arousal associated with the stimuli (Safron et al., 2007). It is not only differences in picture processing due to gender and sexual orientation that play a role in the study of human sexuality. An individual's sexual preferences are also of importance. Findings suggest that one's interest and response to visual sexual stimuli are also dependent upon the activities and situations depicted (Rupp & Wallen, 2009; Stoléru et al., 2012; Wierzba et al., 2015). Thus, in order to dissociate the influence of gender from sexual orientation and sexual preferences it seems necessary to perform counter-balanced studies involving different types of experimental material that appeal to different groups of individuals at a comparable level. Also, individual sexual preferences of participants related to sexual activities and practices have not been considered in previously available stimuli sets.

The current study seeks to further fill important gaps in the literature by examining sexual interests from a broader perspective as sexual attraction, sexual behavior, and one’s sexual orientation identity are all measures of sexual orientation that identify distinct, albeit overlapping, populations (Sell, 2007). Laumann et al. (2000) showed that 8.6–10% of individuals reported some same-sex sexuality, 75–88% reported same-sex sexual desire, 41–52% reported some same-sex sexual behavior, but only 16–27% reported a lesbian or gay identity. Also, in a young Spanish sample, a wide variability in terms of sexual orientation has been reported, with a significant percentage of young people (14.6%) considering themselves homosexual and bisexual and an even higher percentage (35.1%) whose sexual attraction is not directed exclusively towards the other sex (Ballester-Arnal & Gil-Llario, 2016). People who self-identified as heterosexual often experience same-sex attraction, fantasies, desires, and behavioral intentions (Nebot-Garcia et al., 2022). Additionally, when adolescents were asked to define sexual orientation, they did not perceive sexual behavior and self-identification as necessarily relevant but rather the individual's sexual attraction (Friedman et al, 2004). Within this study, participants were not divided into categories according to their self-identified sexual orientation but according to their sexual attraction to best account for sexual interests and not sexual identity, which could be further influenced by social norms and stigma (Grubbs et al., 2019; Schmitt & Fuller, 2015; Walters & Spengler, 2016).

Overall, this research aimed to offer a new standardized set of visual stimuli for emotion and sexuality research, the Explicit Pornographic Picture Set (EPPS), to address the above-mentioned limitations and ensure both experimental and clinical future research in controllable settings. This collection of contemporary pornographic images was selected to represent a wide range of explicit content suitable for research on women and men with exclusive opposite- and same-sex sexual attraction including a hybrid approach of dimensional and discrete assessment of normative affective ratings of valence, arousal, and dominance, as well as disgust and ethical and moral acceptance. This hybrid approach derives from the two predominant conceptualizations present in the study of human emotion, the dimensional and the discrete emotion models related to the assessment of affective responses (Harmon-Jones et al., 2017). The dimensional perspective assumes that emotions can be described in combinations of fundamental dimensions, such as hedonic valence, arousal, and dominance (Lang et al., 1993). The discrete or categorical model states that emotion space is composed of a circumscribed collection of different emotions (Barrett et al., 2007). Among others, disgust and morality have both been named important in human sexuality, especially in the perception and emotional processing of visual sexual stimuli (Andrews et al., 2015; Stark et al. 2005). The explicit content shall reflect a broad variety of content accounting for different sexual practices typically displayed on online pornography platforms, which are also popular in offline sexual encounters such as masturbation, oral, vaginal, anal, group sex and paraphiliac scenes of submission, sexual violence, and fetishes (Hald & Štulhofer, 2016; Herbenick et al., 2022a, b; Richters et al., 2006). Additionally, physical picture properties of EPPS stimuli will be presented since previously available affective or sexual stimuli were often not fully specified and their technical parameters not reported. This is an important contribution to prior literature as such technical features are known to influence the processing of images and hence should be already considered in the process of stimuli selection – especially in assessments of eye-tracking, functional magnetic resonance imaging (fMRI), magnetoencephalography (MEG), and electroencephalography (EEG) (Knebel et al., 2008; Lakens et al., 2013).

For the aforementioned reasons, this investigation had the following objectives: (1) Standardization of the EPPS, including the description of the distribution of the affective ratings. (2) Exploring the influence of gender and sexual attraction on the subjective evaluation of different pornographic stimuli categories. Normative ratings corresponding to diverse content categories of opposite- and same-sex female and male pornography performers will be compared for each group of participants of women with exclusive opposite-sex attraction, women with same-sex attraction, men with exclusive opposite-sex attraction and men with same-sex attraction. We hypothesized to find significant differences in the affective ratings based on the content of different sexual practices, shown opposite- and same-sexes of the pornography performers, and the gender and exclusive opposite- versus same-sex sexual attraction of the observers. Differences in line with the observer's sexual attraction were expected. For instance, individuals with same-sex interests would likely rate same-sex pornographic content of their interest as more emotionally arousing, more pleasant, less disgusting, as well as with higher ratings for dominance, and moral and ethical acceptance (Rupp & Wallen, 2009; Stoléru et al., 2012; Wierzba et al., 2015). (3) Testing the internal reliability of the affective ratings. (4) Reporting the physical properties (width, height, luminance, contrast, complexity, entropy, and color composition) of EPPS pictures, allowing for a more accurate selection of physically matching stimuli.

## Methods

### Participants

A sample of 333 individuals participated in the study. For the purpose of the standardization process, the participants were divided into groups according to their gender (W-women, M-men) and exclusive opposite-sex attraction (OA) or same-sex attraction (SA). Whereby a minimum number of ten participants per group (M-OA, M-SA, W-OA, W-SA) as raters per stimulus was targeted. This sample size of raters per stimulus has been recognized as an appropriate size in other standardization processes (Cui et al., 2021; Daoultzis & Kordoutis, 2022; Diconne et al., 2022).

Participants self-reported their gender, and the following analyses included those who identified as either men or women. Sexual attraction was assessed using Kinsey's (1948) Spanish adapted question asking, "which of the following statements best describes to whom you are sexually attracted?". Participants then responded on a Likert-type scale ranging from 1 = *I am only attracted to the other sex* to 7 = *I am only attracted to my own sex*. There was also another option reserved for asexual people: *I am not attracted to either sex*. Individuals who described themselves as asexual related to their sexual orientation, transsexual, or non-binary related to their gender identity had to be excluded from the further analysis (*n* = 14) in order to compare M-OA, M-SA, W-OA, and W-SA. Individuals who described themselves as only attracted to the other sex according to Kinsey's (1948) question are followingly referred to as women or men with exclusive opposite-sex attraction (W-OA, M-OA), and those who were also attracted to their own sex are referred to as women and men with same-sex attraction (W-SA, M-SA). Thus, from the included participants, those who selected questionnaire option 1 = *I am only attracted to the other sex* according to Kinsey's (1948) question are followingly referred to as women or men with exclusive opposite-sex attraction. Those who have described themselves according to Kinsey's (1948) question as also being attracted to their own sex, respectively, are referred to as women or men with same-sex attraction. Eventually, ratings from a total of 319 participants (44 M-OA, 47 M-SA, 90 W-OA, 138 W-SA) older than 18 years (*M*_age_ = 22.66, *SD*_age_ = 4.66) were considered for the standardization. Post hoc analyses revealed that men with same-sex attraction (M-SA; *M*_age_ = 26.04, *SD*_age_ = 7.40) were significantly older than M-OA *(M*_age_ = 23.18, *SD*_age_ = 3.68, *p* = 0.012), W-OA (*M*_age_ = 21.56, *SD*_age_ = 4.06, *p* < 0.001), and W-SA *(M*_age_ = 22.06, *SD*_age_ = 3.44, *p* < 0.001). Most of the participants (88.25%) were students or graduates from different faculties and departments of various universities in Spain. The other 11.75% reported post-compulsory secondary education. No significant differences were found regarding levels of education between W-OA, M-OA, W-SA, and M-SA participants (*Χ*^2^(3) = 4.408, *p* = 0.221).

Several recruitment channels were used, including mass mailings organized by non-profit organizations, as well as social media and personal invitations of students at the faculties of the Universities of Jaén or Jaume I, Spain. The procedures used in this study adhere to the tenets of the Declaration of Helsinki. Approval was obtained from the ethics committee of the Universitat Jaume I (Castelló de la Plana, Spain).

### Stimulus material

Explicit pornographic pictures (*N* = 1123) were gathered through an extensive online web search and a wide spectrum of pornographic scenarios was collected and eventually categorized by six experts in the field of sexuality according to the sexual practices and the sexes of the pornography performers depicted using a forced-decision task to avoid blended categories of pornographic images. Pictures were only included in the standardization process, if there was total agreement among experts related to the offered categories of masturbation (M), oral sex (O), vaginal sex (V), anal sex (A), group sex (G), and paraphilia (P).

After the categorization by experts in the field, for this first standardization, four sets of each 48 sexually explicit pornographic images from the first time sought explicit pornographic pictures (*N* = 1123) and the 24 IAPS^1^ (Lang et al., 1999) control stimuli were used. Since the IAPS constitutes a well-established and world-wide used resource of standardized pictorial stimuli for emotion research, images of mutilation (unpleasant), sports/adventure (pleasant) and neutral people (neutral) were presented to be able to employ and compare control stimuli of affective value without sexual content (Lang et al., 1999; Lang & Bradley, 2007). Pictures from the IAPS were selected based on the normative values for affective valence and arousal in Spanish samples (Moltó et al., 1999, 2013; Vila et al., 2001). IAPS normative affective ratings for each category were as follows: unpleasant (valence: *M* = 1.88; *SD* = 0.34; arousal: *M* = 7.43; *SD* = 0.25), pleasant (valence: *M* = 6.78; *SD* = 0.50; arousal: *M* = 7.33; *SD* = 0.48), and neutral pictures (valence: *M* = 5.19; *SD* = 0.51; arousal: *M* = 3.49; *SD* = 0.32).

The pornographic images included here differ in the types of sexual content shown, providing a diversity of highly explicit scenes, as well as diversity related to depicted sexes of the pornography performers, offering same-sex, opposite-sex, solo-sex, and group-sex scenarios. Like Hald (2006) categorized any kind of material aiming at creating or enhancing sexual feelings or thoughts in the recipient and, at the same time, containing explicit exposure and/or descriptions of the genitals or clear and explicit sexual acts as pornographic, pictures of M, O, V and A were labeled as explicit pornographic pictures. The categories of M, O and A provide same-sex and opposite-sex imagery. Additionally, this set offers an extra category of pictures of G scenarios as this type of content is also considered to be a very popular type of pornography (Hald & Štulhofer, 2016). Furthermore, scenarios of dominance/submission, sexual violence (sadomasochism, violent sex, aggression, bondage), and fetishes (including latex) were included and categorized under the extra category of paraphilia pornography (P). In a few cases, the categories of M and P only showed single individuals.

**Fig. 1:**
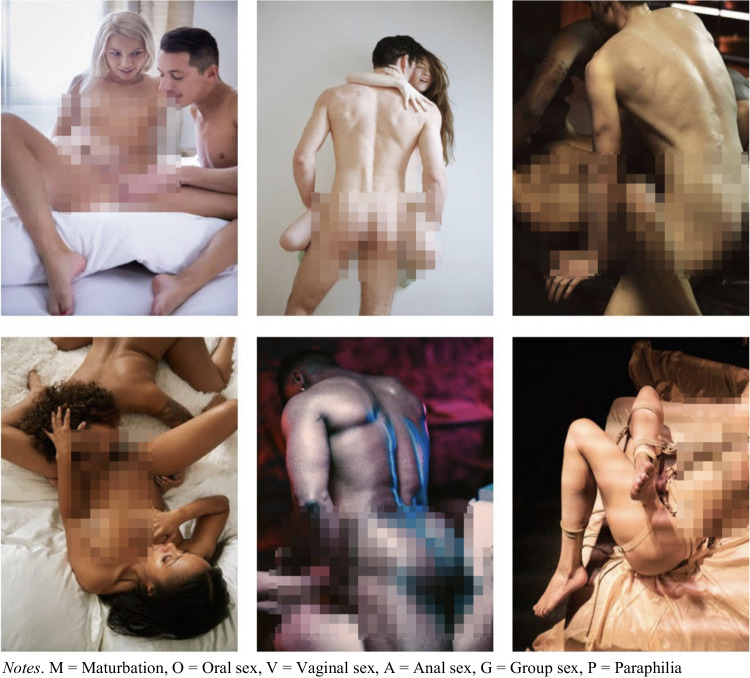
Pixelated example images of the EPPS categories showing sexual practices of M, O, V, A, G, P

All sexually explicit pictures were resized to 1600×1200 or 1200×1600 pixels size and were granted access to the project by the pornography producers and distributors ErikaLust film productions or AmazingContent or were acquired from amateur (flickr) pornography platforms. Permission to use these images for research purposes has been granted by the aforementioned pornography producers and distributors. This approach was chosen to limit the possibility of including nonconsensual pornography, which is a significant ethical issue when working with freely available online sexual stimuli (Katz et al., 2023). Examples of the explicit pornographic pictures of the M, O, V, A, G, P categories are displayed in Fig. 1.

## Procedure

Due to the sanitary health crisis inflicted by COVID-19, the assessment procedure took place via online meetings using Google Meet and Qualtrics XM. The participants worked on their private devices (PC, laptop, or tablet). Sessions lasted approximately 90 min. Each experimental session was conducted either in groups of 2–10 participants or in individual sessions, if it was requested by the participant.

Before the participants could start the study, they had to complete a short introduction and practice phase via Google Meet given by two experimental instructors in order to make sure that the instructions regarding the affective ratings were understood. Three practice pictures were viewed prior to the experimental ratings in order to provide participants with a range of the types of content that will be presented, as well as to anchor the affective rating scales. During the whole procedure, participants were able to ask the two instructors via Google Meet for private assistance when in doubt. Written consent was obtained from each participant and the possibility to quit the experiment at any point without stating reasons was ensured. The full text of the Spanish instructions and its English translation are enclosed in the supplementary materials.

Each participant was presented with 72 pictures equaling one set of images, whereby to date in total, four independent sets of explicit pornographic pictures were standardized. The stimuli consisted of equal-sized (eight images each) categories of sexual practices: masturbation (M), oral sex (O), vaginal sex (V), anal sex (A), group sex (G), paraphilia (P). As control, images of mutilation, sports and neutral people of the IAPS were included (eight per category) into the standardization process. Stimuli were presented in randomized order using the Qualtrics randomization tool.

**Fig. 2:**
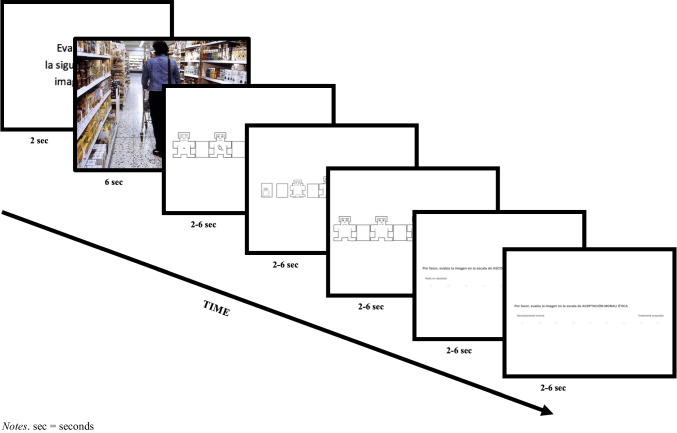
Schematic representation of a trial with a neutral IAPS control stimulus followed by the  assessed affective rating scales

The images were assessed one at a time. A single picture was displayed for 6 s, and then on the following pages adapted versions of the nine-point Self-Assessment Manikin (Bradley & Lang, 1994) rating scales for the dimensional affective ratings of valence, arousal and dominance were presented in a randomized order to avoid halo effects. Followingly nine-point Likert rating scales for the discrete emotional categories of disgust as well as moral and ethical acceptance were presented also in a randomized order. The disgust scale ranged from 1 – this picture is nothing at all, to 9 – this picture is extremely disgusting. The moral and ethical acceptance scale ranged from 1 – absolutely immoral, to 9 – totally acceptable. Figure 2 shows a schematic representation of this sequence with an exemplar of a neutral IAPS image and the adapted versions of the SAM for valence, arousal, and dominance ratings, as well as the ratings scales used to assess disgust and ethical and moral acceptance. Participants were asked to give an immediate, spontaneous reaction to the seen pictures. For 6 s, each rating scale was available before skipping to the next. After 2 s, additionally, participants also had the option to click on a next button in order to see the next rating scale. As soon as the participants finished the ratings of one picture, a new screen with the subsequent randomized picture was displayed.

After the affective ratings task participants were presented with questionnaires, as well as sexual behavior questions, which will be presented elsewhere. On average the participants needed 56.1 min (*SD* = 8.87) to finish the online part of the study.

### Statistical analysis

Data were analyzed using the statistics program JMP 17.0 and RStudio Desktop (RStudio Inc., Boston, MA, USA). The significance threshold was set to α = 0.05, thus the cut-off for statistical significance was *p* < 0.05.

## Results

### Affective ratings

Ratings from 67.7 participants on average were collected for each picture (*n* = 77 for Set 1, *n* = 82 for Set 2, *n* = 94 for Set 3, *n* = 66 for Set 4). Mean and standard deviation for the ratings in valence, arousal, dominance, disgust, and moral and ethical acceptance were calculated for each individual picture, for the overall sample and split for women and men with exclusive opposite-sex attraction vs. same-sex attraction separately (W-OA, W-SA, M-OA, M-SA). This data can be found in the supplementary material.

Furthermore, to analyze relationships between the affective ratings of the explicit pornographic stimuli, we first employed bivariate Pearson correlations between valence and arousal. A significant positive correlation between valence and arousal ratings (*r*(190) = .66, *p* < .0001) was found. Valence also correlated positively with dominance (*r*(190) = .71, *p* < .0001) and ratings of moral and ethical acceptance (*r*(190) = .77, *p* < .0001), but negatively with disgust (*r*(190) = – .62, *p* < .0001). Similarly, arousal was positively related with dominance (*r*(190) = .37, *p* < .0001) and moral and ethical acceptance (*r*(190) = .23, *p <* .0001), but negatively with disgust (*r*(190) = – .30, *p <* .0001). Further significant negative correlations were found between ratings of disgust and moral and ethical acceptance (*r*(190) = – .54, *p <* .0001). These analyses were performed separately for each participant group. As observed in Table 1, the results were similar in all groups, except for the relationship between arousal and dominance ratings, which only was statistically significant in the M-OA and W-SA groups. Also, the correlations between arousal and moral and ethical acceptance did not reach the significance level in W-SA but all the other groups.

**Table 1:** Correlation matrix of valence, arousal, dominance, disgust, and moral and ethical acceptance ratings split for W-OA, W-SA, M-OA, M-SA participant groups

		M-OA				M-SA				W-OA				W-SA			
		1	2	3	4	1	2	3	4	1	2	3	4	1	2	3	4
1.Valence																	
2.Arousal		.89***				.65***				.59***				.58***			
3.Dominance		.63***	.56***			.53***	.11			.48***	.13			.67***	.41***		
4.Disgust		– .92***	– .76***	– .57***		– .86***	– .49***	– .64***		– .81***	– .22**	– .49***		– .86***	– .27***	– .57***	
5.Moral and ethical acceptance		.64***	.54***	.51***	– .66***	.74***	.27***	.52***	– .76***	.75***	.15*	.46***	– .75***	.75***	.13	.50***	– .70***

M-OA = Men with exclusive opposite-sex attraction, M-SA = Men with same-sex attraction, W-OA = Women with exclusive opposite-sex attraction, W-SA = Women with same-sex attraction.

^*^p < 0.05, **p < 0.01, ***p < 0.001

In addition, the relationships between the affective ratings of the IAPS control pictures were analyzed with bivariate Pearson correlations. A significant negative correlation was found (*r*(190) = – .51, *p* = .01) between valence and arousal. Nevertheless, the IAPS ratings of valence correlated, like the EPPS stimuli, positively with dominance (*r*(190) = .86, *p* < .0001) and moral and ethical acceptance (*r*(190) = .98, *p* < .0001), but negatively with disgust (*r*(190) = – .98, *p* < .0001). For the IAPS pictures, negative relationships between arousal and dominance (*r*(190) = – .86, *p* < .0001) and moral and ethical acceptance (*r*(190) = – .66, *p* < .0001) were found. Moreover, IAPS ratings of arousal correlated positively with disgust (*r*(190) = .65, *p* < .0001). Again, similar to EPPS images, significant negative correlations were found for the IAPS control stimuli between disgust and moral and ethical acceptance (*r*(190) = – .99, *p* < .0001).

### Gender, sexual attraction, and stimuli content

**Fig. 3:**
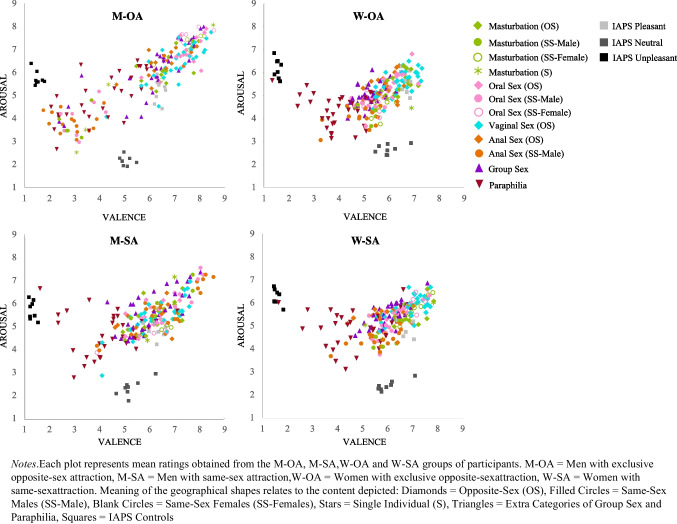
Distribution of the mean ratings obtained for EPPS and IAPS control images in the affective space of valence and arousal

Furthermore, the affective ratings to the M, O, V, A, G, P sexual practice categories of opposite- and same-sex content have been compared in the W-OA, W-SA, M-OA, M-SA participant groups. Figure 3 shows the affective space formed by averaged valence and arousal ratings to the different sexual practices and IAPS control pictures, for W-OA, W-SA, M-OA, M-SA separately. The scatterplots of this study show a particular distribution of the pornographic content as most of the pornographic images score high in valence and arousal. Including the IAPS control images, a curvilinear pattern of distribution emerged for all participant groups. In the scatterplots is also included the distribution of the different sexual practices (M, O, V, A, G, P) by opposite- and same-sex (male, female) content, showing, for example, that the high arousal and high valence quadrant presented for W-OA, W-SA and M-OA a relatively higher number of M, O and V, G pictures, but for M-SA this quadrant also included a relatively bigger number of A same sex male images. Further, the space of lower valence and arousal scores presented a relatively high number of P content for all participants.

**Fig. 4:**
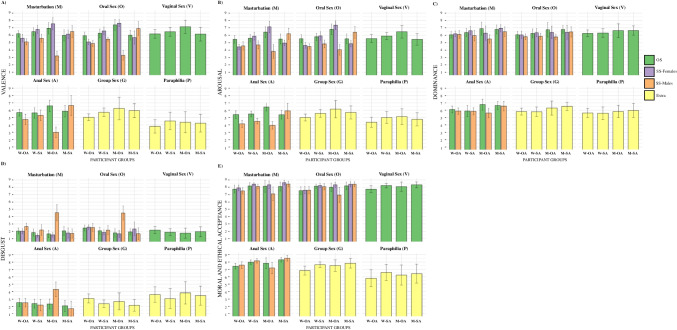
Bar charts of the affective ratings of shown sexual practices and sexes by gender and sexual attraction groups of the affective ratings of valence, arousal, dominance, disgust, and moral and ethical acceptance

Three-way ANOVAs were performed with these mean ratings to evaluate the effects of participant groups defined by gender and sexual attraction (W-OA, W-SA, M-OA, M-SA), and stimuli content of shown sexual practices (M, O, V, A, G, P) and sexes (opposite-sex, same-sex male, same-sex female) on the affective ratings of valence, arousal, dominance, disgust, and moral and ethical acceptance. The few M images (*N* = 3) that only showed single individuals were added to either the content category of SS-Females when a female single individual, or to the content category of SS-Males when a male single individual, was depicted. Regarding depicted sexes, the images of group sex (G) and paraphilia (P) were categorized as extra categories. Figure 4 shows these mean values of the averaged affective ratings to the EPPS pictures of the different shown sexual practices and shown sexes for the participant groups of W-OA, W-SA, M-OA, M-SA. For valence, main effect of participant groups (*F*(3, 720) = 21.51, *p* < 0.001, η2 = 0.03), shown sexual practices (*F*(5, 718) = 109.76,* p* < 0.001, η2 = 0.11), and shown sexes (*F*(2, 721) = 74.29, *p* < 0.001, η2 = 0.08) were found. There was no statistically significant three-way interaction for valence, but significant two-way interactions between participant groups and shown sexual practices (*F*(15, 708) = 5.89,* p* < 0.001, η2 = 0.01), and between participant groups and shown sexes (*F*(6, 717) = 53.18, *p* < 0.001, η2 = 0.17). Also, for arousal, main effects of participant groups (*F*(3, 720) = 52.05, *p* < 0.001, η2 = 0.10), shown sexual practices (*F*(5, 718) = 36.16,* p* < 0.001, η2 = 0.06), and shown sexes (*F*(2, 721) = 74.59, *p* < 0.001, η2 = 0.10) were found. There was also no statistically significant three-way interaction for arousal, but significant two-way interactions between participant groups and shown sexual practices (*F*(15, 708) = 3.49,* p* < 0.001, η2 = 0.01), and between participant groups and shown sexes (*F*(6, 717) = 48.46, *p* < 0.001, η2 = 0.19). For dominance, main effects of participant groups (*F*(3, 720) = 27.39, *p* < 0.001, η2 = 0.08), shown sexual practices (*F*(5, 718) = 18.53,* p* < 0.001, η2 = 0.02), and shown sexes (*F*(2, 721) = 16.08, *p* < 0.001, η2 = 0.03) were found. There was also no statistically significant three-way interaction for dominance, but a significant two-way interaction between participant groups and shown sexes (*F*(6, 717) = 5.04, *p* < 0.001, η2 = 0.03). Analysis of disgust ratings showed main effects of participant groups (*F*(3, 720) = 21.41, *p* < 0.001, η2 = 0.05), shown sexual practices (*F*(5, 718) = 59.90,* p* < 0.001, η2 = 0.07), and shown sexes (*F*(2, 721) = 23.30, *p* < 0.001, η2 = 0.03). Three-way interaction for disgust did not reach the significance level, but the two-way interactions between participant groups and shown sexual practices (*F*(15, 708) = 3.38,* p* < 0.001, η2 = 0.01), between participant groups and shown sexes (*F*(6, 717) = 24.90, *p* < 0.001, η2 = 0.11), and between shown sexual practices and sexes (*F*(3, 720) = 2.64, *p* = 0.049, η2 = 0.01) were significant. For ratings of moral and ethical acceptance, main effects of participant groups (*F*(3, 720) = 42.17, *p* < 0.001, η2 = 0.08), shown sexual practices (*F*(5, 718) = 129.52,* p* < 0.001, η2 = 0.12), and shown sexes (*F*(2, 721) = 6.18, *p* < 0.001, η2 = 0.01) were found. There was also no statistically significant three-way interaction for moral and ethical acceptance, but a significant two-way interaction between participant groups and shown sexes (*F*(6, 717) = 24.90, *p* < 0.001, η2 = 0.02).

Additionally, to offer a more in-depth analysis of each image category related to the participants gender and sexual attraction, the mean scores for the affective ratings of each image were compared within each sexual practice split by shown sexes between W-OA, W-SA, M-OA, M-SA participant group using one-way analyses of variance and pairwise multiple-comparisons tests, with Tukey HSD post hoc tests and Cohen’s *d* effect sizes (see Table 2). Regarding affective ratings of valence, W-OA, W-SA, M-OA, M-SA participants only showed no significant differences related to the P category (*p* = 0.109). Further, no significant differences between W-OA, W-SA, M-OA, M-SA participants were observed in the dominance ratings related to same-sex female M (*p* = 0.103), same-sex female O (*p* = 0.728), V (*p* = 0.05) and P (*p* = 0.205) images. Regarding disgust ratings the W-OA, W-SA, M-OA, M-SA participants only showed no significant differences related to the opposite-sex M (*p* = 0.079), opposite-sex A (*p* = 0.281) and P (*p* = 0.124) images. Also, for ratings of moral and ethical acceptance no significant differences were found between W-OA, W-SA, M-OA, M-SA participants in opposite-sex M (*p* = 0.21) and P (*p* = 0.06) image content.

**Table 2:** Comparison of valence, arousal, dominance, disgust, and moral and ethical acceptance ratings for W-OA, W-SA, M-OA, M-SA participants

		M-OA		M-SA		W-OA		W-SA		ANOVA (*df*; *F*; *p; η2*)	Post hoc *t t*est Tukey HSD (Cohen's *d*)
		Mean	*SD*	Mean	*SD*	Mean	*SD*	Mean	*SD*		
Valence											
	M-OS	6.94	0.77	5.99	0.75	6.20	0.43	6.48	0.52	3; 6.708; < 0.001***; 0.25	M-OA>M-SA*** (*d* = 1.25), W-OA** (*d* = 1.19)
	M-SS-Female	7.56	0.82	6.15	0.56	5.62	0.51	6.80	0.53	3; 14.62; < 0.001***; 0.61	M-OA>M-SA*** (*d* = 2.00), W-OA*** (*d* = 2.84); W-SA>W-OA** (*d* = 2.27)
	M-SS-Male	3.22	0.63	6.50	0.82	5.12	0.42	5.61	0.54	3; 40.03; < 0.001***; 0.81	M-SA>M-OA*** (*d* = 4.49), W-OA*** (*d* = 2.13), W-SA* (*d* = 1.29); M-OA<W-OA*** (*d* = – 3.54), W-SA*** (*d* = – 4.06)
	O-OS	7.41	0.73	6.02	0.65	5.94	0.49	6.27	0.57	3; 19.52; < 0.001***; 0.49	M-OA>M-SA*** (*d* = 2.01), W-OA*** (*d* = 2.36), W-SA*** (*d* = 1.73)
	O-SS-Male	3.29	0.66	6.91	0.94	4.89	0.41	5.48	0.26	3; 45.96; < 0.001***; 0.83	M-SA>M-OA*** (*d* = 4.45), W-OA*** (*d* = 2.79), W-SA*** (*d* = 2.08); M-OA<W-OA*** (*d* = – 2.90), W-SA*** (*d* = – 4.33)
	O-SS-Female	7.64	0.47	5.71	0.88	5.11	0.36	6.58	0.52	3; 50.06; < 0.001***; 0.75	M-OA>M-SA*** (*d* = 2.74), W-OA*** (*d* = 6.04), W-SA** (*d* = 2.13); W-SA>M-SA* (*d* = 1.21), W-OA*** (*d* = 3.27)
	V	7.14	0.89	6.15	0.92	6.18	0.57	6.45	0.61	3; 11.75; < 0.001***; 0.22	M-OA>M-SA*** (*d* = 1.10), W-OA*** (*d* = 1.29), W-SA** (*d* = 0.89)
	A-OS	6.59	0.78	5.85	0.78	5.66	0.48	5.62	0.70	3; 6.735; < 0.001***; 0.25	M-OA>M-SA* (*d* = 0.95), W-OA** (*d* = 1.44), W-SA** (*d* = 1.31)
	A-SS-Male	2.98	0.72	6.63	1.36	4.74	0.70	5.30	0.71	3; 43.53; < 0.001***; 0.69	M-SA>M-OA*** (*d* = 3.36), W-OA*** (*d* = 1.75), W-SA*** (*d* = 1.24); M-OA<W-OA*** (*d* = – 2.46), W-SA*** (*d* = – 3.23)
	G	6.24	1.54	5.97	0.97	4.99	0.46	5.69	0.59	3; 8.86; < 0.001***; 0.18	W-OA<M-OA*** (*d* = – 1.06), M-SA** (*d* = – 1.22), W-SA* (*d* = – 1.31)
	P	4.35	1.43	4.23	1.19	3.79	0.91	4.52	1.19	3; 2.202; 0.109; 0.06	-
Arousal											
	M-OS	6.44	0.71	5.50	0.67	5.46	0.52	5.62	0.50	3; 9.157; < 0.001***; 0.31	M-OA>M-SA*** (*d* = 1.35), W-OA*** (*d* = 1.57), W-SA** (*d* = 1.33)
	M-SS-Female	7.14	0.77	4.96	0.39	4.45	0.37	5.88	0.51	3; 39.01; < 0.001***; 0.81	M-OA>M-SA*** (*d* = 3.58), W-OA*** (*d* = 4.46), W-SA*** (*d* = 1.93); W-SA>M-SA*** (*d* = 2.01), W-OA** (*d* = 3.19)
	M-SS-Male	3.83	0.93	6.21	0.75	4.58	0.57	4.72	0.55	3; 15.32; < 0.001***; 0.62	M-SA>M-OA*** (*d* = 2.80), W-OA*** (*d* = 2.43), W-SA** (*d* = 2.26)
	O-OS	6.80	0.72	5.53	0.65	5.54	0.54	5.80	0.48	3; 15.84; < 0.001***; 0.44	M-OA>M-SA*** (*d* = 1.85), W-OA*** (*d* = 1.98), W-SA*** (*d* = 1.63)
	O-SS-Male	4.06	0.74	6.42	0.80	4.49	0.36	4.85	0.51	3; 21.27; < 0.001***; 0.70	M-SA>M-OA*** (*d* = 3.05), W-OA*** (*d* = 3.10), W-SA*** (*d* = 2.34)
	O-SS-Female	7.37	0.59	4.88	0.43	4.65	0.38	5.91	0.56	3; 50.06; < 0.001***; 0.84	M-OA>M-SA*** (*d* = 4.87), W-OA*** (*d* = 5.52), W-SA*** (*d* = 2.53); W-SA>M-SA*** (*d* = 2.09), W-OA** (*d* = 2.65)
	V	6.45	0.84	5.46	0.76	5.56	0.53	5.89	0.49	3; 15.38; < 0.001***; 0.27	M-OA>M-SA*** (*d* = 1.29), W-OA*** (*d* = 1.33), W-SA*** (*d* = 0.87)
	A-OS	6.44	0.54	5.38	0.58	5.40	0.51	5.48	0.38	3; 16.07; < 0.001***; 0.45	M-OA>M-SA*** (*d* = 1.88), W-OA*** (*d* = 1.97), W-SA*** (*d* = 2.04)
	A-SS-Male	3.93	0.50	5.91	1.01	4.15	0.49	4.49	0.38	3; 30.43; < 0.001***; 0.60	M-SA>M-OA*** (*d* = 2.47), W-OA*** (*d* = 2.21), W-SA*** (*d* = 1.85)
	G	6.20	1.16	5.75	0.91	5.01	0.46	5.57	0.51	3; 10.6; < 0.001***; 0.20	W-OA<M-OA*** (*d* = – 1.27), M-SA** (*d* = – 0.98), W-SA* (*d* = – 1.12); W-SA<M-OA* (*d* = – 0.65)
	P	5.12	1.08	4.76	0.89	4.36	0.68	4.99	0.80	3; 4.473; < 0.001***; 0.10	W-OA<M-OA** (*d* = – 0.85), W-SA** (*d* = – 0.84)
Dominance											
	M-OS	6.89	0.89	6.79	0.60	6.07	0.43	6.37	0.54	3; 5.8; < 0.01**; 0.22	W-OA<M-OA** (*d* = – 1.19), M-SA* (*d* = – 1.39)
	M-SS-Female	6.29	0.96	6.94	0.48	6.20	0.37	6.61	0.56	3; 2.26; 0.103; 0.19	-
	M-SS-Male	5.51	0.56	6.49	0.66	6.12	0.52	5.95	0.70	3; 3.433; < 0.05*; 0.27	M-SA>M-OA* (*d* = 1.59)
	O-OS	6.75	0.98	6.78	0.53	6.07	0.45	6.26	0.60	3; 4.44; < 0.01**; 0.18	W-SA<M-OS* (*d* = – 0.61), M-SA* (*d* = – 0.61)
	O-SS-Male	5.78	0.39	6.46	0.69	5.80	0.39	5.86	0.42	3; 3.538; < 0.05*; 0.27	M-SA>M-OA* (*d* = 1.22)
	O-SS-Female	6.39	0.85	6.42	0.94	6.05	0.47	6.35	0.54	3; 0.437; 0.728; 0.04	-
	V	6.63	0.92	6.64	0.63	6.26	0.45	6.31	0.57	3; 2.963; 0.05; 0.07	-
	A-OS	6.77	0.76	6.63	0.56	6.09	0.46	5.88	0.75	3; 6.788; < 0.001***; 0.25	W-OA<M-OA* (*d* = – 1.07); W-SA<M-OA** (*d* = – 1.17), M-SA** (*d* = – 1.12)
	A-SS-Male	5.61	0.64	6.55	0.65	5.88	0.43	5.84	0.47	3; 8.504; < 0.001***; 0.30	M-SA>M-OA*** (*d* = 1.46), W-OA** (*d* = 1.22), W-SA** (*d* = 1.24)
	G	6.28	0.99	6.49	0.51	5.80	0.47	5.76	0.67	3; 9.008.; < 0.001***; 0.18	W-OA<M-OA* (*d* = – 0.64), M-SA*** (*d* = – 1.36); W-SA<M-OA* (*d* = – 0.62), M-SA*** (*d* = – 1.21)
	P	5.82	0.84	5.96	0.95	5.61	0.63	5.58	0.86	3; 1.55; 0.205; 0.04	-
Disgust											
	M-OS	1.65	0.49	2.06	0.60	2.04	0.41	1.83	0.49	3; 2.38; 0.079; 0.11	-
	M-SS-Female	1.51	0.30	1.78	0.68	2.03	0.33	1.44	0.22	3; 3.215; < 0.05*; 0.26	W-OA>W-SA* (*d* = 2.07)
	M-SS-Male	4.54	1.12	1.71	0.62	2.66	0.44	2.19	0.72	3; 20.79; < 0.001***; 0.69	M-OA>M-SA*** (*d* = 3.12), W-OA*** (*d* = 2.20), W-SA*** (*d* = 2.50)
	O-OS	1.79	0.57	1.92	0.43	2.46	0.40	2.08	0.49	3; 5.91; < 0.01***; 0.23	W-OA>M-OA** (*d* = 1.37), M-SA* (*d* = 1.28)
	O-SS-Male	4.50	0.97	1.68	0.80	2.52	0.55	2.17	0.64	3; 21.41; < 0.001***; 0,70	M-OA>M-SA*** (*d* = 3.17), W-OA*** (*d* = 2.51), W-SA*** (*d* = 2.84)
	O-SS-Female	1.66	0.46	2.33	0.92	2.58	0.31	1.85	0.35	3; 4.472; < 0.05*; 0.32	W-OA>M-OA* (*d* = 2.34)
	V	1.76	0.68	1.95	0.68	2.17	0.50	1.88	0.47	3; 2.752; < 0.05*; 0.06	W-OA>M-OA* (*d* = 0.67)
	A-OS	2.34	0.68	2.08	0.74	2.53	0.57	2.39	0.61	3; 1.305; 0.281; 0.06	-
	A-SS-Male	4.30	0.98	1.69	1.03	2.50	0.57	2.19	0.78	3; 27.92; < 0.001***; 0.58	M-OA>M-SA*** (*d* = 2.60), W-OA*** (*d* = 2.24), W-SA*** (*d* = 2.38); W-SA>M-SA* (*d* = 0.55)
	G	2.68	1.15	2.15	0.80	3.11	0.61	2.40	0.54	3; 8.03; < 0.001***; 0.16	W-OA> M-SA*** (*d* = 1.31), W-SA** (*d* = 1.27)
	P	3.83	1.49	3.46	1.29	3.61	1.03	3.06	1.34	3; 1.954; 0.124; 0.05	-
Moral and Ethical Acceptance											
	M-OS	8.14	0.81	8.10	0.61	7.76	0.53	8.17	0.46	3; 1.553; 0.21; 0.07	-
	M-SS-Female	8.33	0.51	8.63	0.30	7.92	0.39	8.42	0.17	3; 5.374; < 0.01**; 0.37	W-OA<M-SA** (*d* = – 2.06), W-SA* (*d* = – 1.69)
	M-SS-Male	7.10	0.93	8.43	0.39	7.51	0.43	8.10	0.32	3; 8.673; < 0.001***; 0.48	M-SA>M-OA*** (*d* = 1.86), W-OA* (*d* = 2.25); M-OA<W-SA** (*d* = – 1.44)
	O-OS	7.99	0.58	8.19	0.52	7.55	0.52	8.14	0.29	3; 5.684; < 0.01**; 0.22	W-OA<M-SA** (*d* = – 1.23), W-SA** (*d* = – 1.39)
	O-SS-Male	6.94	1.02	8.41	0.34	7.62	0.52	8.08	0.43	3; 8.021; < 0.001***; 0.46	M-OA<M-SA*** (*d* = – 1.93), W-SA** (*d* = – 1.45)
	O-SS-Female	8.33	0.35	8.40	0.35	7.62	0.50	8.23	0.27	3; 7.281; < 0.001***; 0.44	W-OA<M-OA** (*d* = – 1.67), M-SA** (*d* = – 1.89), W-SA* (*d* = – 1.53)
	V	8.09	0.62	8.33	0.39	7.75	0.48	8.23	0.30	3; 15.38; < 0.001***; 0.19	W-OA<M-OA* (*d* = – 0.60), M-SA*** (*d* = – 1.34), W-SA*** (*d* = – 1.21)
	A-OS	7.83	0.74	8.31	0.31	7.43	0.39	7.99	0.29	3; 9.56; < 0.001***; 0.32	M-SA>M-OA* (*d* = 0.83), W-OA*** (*d* = 2.47); W-SA>W-OA** (*d* = 1.60)
	A-SS-Male	7.19	0.73	8.51	0.38	7.58	0.45	8.16	0.30	3; 22.81; < 0.001***; 0.53	M-SA>M-OA*** (*d* = 2.27), W-OA*** (*d* = 2.24); W-SA>M-OA*** (*d* = 1.73), W-OA** (*d* = 1.51)
	G	7.50	0.78	7.84	0.67	6.81	0.58	7.61	0.37	3; 15.29; < 0.001***; 0.27	W-OA<M-OA*** (*d* = – 0.97), M-SA*** (*d* = – 1.57), W-SA*** (*d* = – 1.58)
	P	6.23	1.36	6.41	1.30	5.79	1.15	6.58	1.10	3; 2.477; 0.06; 0.06	-

M-OA = Men with exclusive opposite-sex attraction, M-SA = Men with same-sex attraction, W-OA = Women with exclusive opposite-sex attraction, W-SA = Women with same-sex attraction. M = Masturbation, O = Oral sex, V = Vaginal Sex, A = Anal Sex, G = Group Sex, P = Paraphilia, OS = Opposite-sex, SS = Same-sex content.

^*^p < 0.05, **p < 0.01, ***p < 0.001

### Reliability of EPPS affective ratings

Regarding the internal consistency of these evaluations, split-half reliability scores (Ruiz-Padial et al., 2021; Wierzba et al., 2015) were calculated. To this end, participants were numbered according to their order of participation, and each sample that evaluated one of the four sets of pictures was split into two groups (i.e., odd vs. even participant numbers). The average ratings for valence, arousal, dominance, disgust, and moral and ethical acceptance were then calculated separately for each picture and within each participant group. To calculate split-half reliability scores, Pearson correlations among these average ratings were analyzed for the two groups of participants of each sample. All correlations were significant (*p* < 0.001), and Spearman–Brown-corrected reliability scores were high for the four sets of pictures (valence: *r* = 0.984, *r* = 0.979, *r* = 0.987, *r* = 0.960; arousal: *r* = 0.953, *r* = 0.945, *r* = 0.958, *r* = 0.904; dominance: *r* = 0.941, *r* = 0.944, *r* = 0.936, *r* = 0.873; disgust: *r* = 0.984, *r* = 0.990, *r* = 0.991, *r* = 0.983; moral and ethical acceptance: *r* = 0.991, *r* = 0.990, *r* = 0.994, *r* = 0.972 for Sets 1, 2, 3, and 4, respectively).

### Physical picture parameters of EPPS stimuli

Physical picture parameters of luminance, contrast, mean channel values in CIE 1976 L*a*b color space, spatial frequency in nine different bands, and JPG size are reported in the supplementary material. The physical picture parameters were calculated using MATLAB (www.mathworks.com). Luminance was defined as the average pixel value of the grayscale version of the images, while contrast consists of the standard deviation of the pixel values of those (Bex & Makous, 2002). Further, the JPEG compression rate of images, and the entropy, which refers to the level of intensity of individual pixels in grayscale images, are considered indicators of image complexity (Donderi, 2006). The JPEG sizes of the color images were determined with a compression quality setting of 80 (on a scale of 1 to 100), indicating the bigger the more complex an image is. In addition, each picture was converted to the CIE L*a*b* color space. The color composition in CIE 1976 L* a* b* space expresses color in three values considering the level of brightness and the values of the four basic colors of human vision (red, green, blue, yellow), and approximates characteristics of the human visual system. The L* dimension corresponds to luminance (range, 0–100), and a* and b* correspond to two chromatic channels ranging from red (positive values) to green (negative values), and from blue (negative values) to yellow (positive values) (Tkalcic & Tasic, 2003).

To analyze whether the average affective ratings of valence and arousal given by the participants for the EPPS images were related to physical picture properties, as seen in other studies (e.g., Bradley & Lang 2000; Carretié et al., 2019), bivariate Pearson correlations were performed. Neither luminance nor contrast or entropy (*p* > .05) were significantly associated with valence and arousal. Regarding L* and a* color space, a significant correlation was only found between arousal and the a* dimension (*r*(190) = .15, *p* = .03). For the b* dimension, significant correlations were found with valence (*r*(190) = .21, *p* = .004) and arousal (*r*(190) = .18, *p* = .01). JPEG size correlated for the EPPS images negatively with valence (*r*(190) = – .23, *p* = .002) and arousal (*r*(190) = – .19, *p* = .01), meaning that less complex images received higher affective valence and arousal ratings.

## Discussion

This paper presents EPPS, a stimuli set of explicit pornographic pictures suitable for experimental research in a wide range of disciplines. The collection is useful for studies with men and women who differ in terms of their sexual attraction to the male or female sex. In this regard, the EPPS differs from all other existing sets of standardized sexual stimuli, which have previously relied on strict categorization into heterosexual and homosexual orientations (Cui et al., 2021; Daoultzis & Kordoutis, 2022; Jacob et al., 2011; Wierzba et al., 2015). This approach was taken because a person's sexual orientation need not be a one-to-one reflection of his or her exclusive sexual preferences for a particular sex at a particular time. Indeed, individuals who describe themselves as heterosexual may nevertheless feel strong sexual attraction towards a person of the same-sex (Katz-Wise & Todd, 2022; VanderLaan et al., 2022). Models trying to define sexual orientation have changed over time. Today, models that attempt to create static categories for sexual orientation are often avoided. Instead, there is increased reliance on more fluid models that allow for changes in sexual orientation over time and that speak to situational flexibility in an individual's sexual responsiveness (Ballester-Arnal & Gil-Llario, 2016). Thus, it has been recognized that changes in sexual desire can be experienced in both the short and long term (Klein, 1978; Klein et al., 1985). Sexual attraction can change as a result of a variety of situational, interpersonal, and social factors (Katz-Wise & Todd, 2022). It is especially important to be aware of this aspect when interested in how initial sexual responses occur and sexual arousal manifests, especially from a physiological point of view. Sexual orientation also reflects the emotional, romantic, or – but not necessarily – sexual attraction that a person feels toward another person (Klein, 1978; Klein et al., 1985). Sexual orientation can thus be understood as a more diverse concept of the self in relation to the unfolding of interpersonal (sexual) relationships, whereas sexual attraction might be closer to the initial sexual responses of sexual arousal. As previously reported, there seems to exist a wide variability in terms of self-reported sexual orientation and sexual attraction (Ballester-Arnal & Gil-Llario, 2016; Laumann et al., 2000), with higher percentages reporting same-sex sexual desires and same-sex sexual attraction, but fewer individuals identify themselves as non-heterosexual. So, asking for a person's *sexual attraction* might be able to better capture these sexual desires – which likely play out also in physiological responses when confronted with sexually interesting stimuli – than asking about *self-reported sexual orientation –* which does not seem to be perceived as sexual attraction that a person feels toward another one. With the EPPS, researchers will have the opportunity to be aware of such potential differences in advance, to have data that address sexual attraction, and to try to minimize romantic and emotional influences of sexual orientation.

The EPPS offers explicit sexual images along with a hybrid approach of dimensional and discrete assessment of normative ratings of valence, arousal, and dominance, as well as disgust and ethical and moral acceptance. Similar to other standardization studies of sexual images there was content that was rated as very positive in terms of these affective ratings by the participants. However, there were also categories of sexual practices which were rated as rather unpleasant by all groups of participants, such as the category of paraphilia stimuli. Like other studies, where applicable, a distinction is made between opposite- and same-sex scenes in the categorization of sexual stimuli (Stoléru et al., 2012; Wierzba et al., 2015). Thereby, further were sexual images identified which were rated as unpleasant by one group of participants but pleasant by another due to the opposite- and same-sex content shown. For example, those depicting male couples were rated as least pleasant, arousing, able to maintain control and ethical and morally acceptable but most disgusting by men with opposite sex attraction but perceived in the opposite way by men with same sex attraction. Men with opposite sex attraction seem to show strong preferences for opposite-sex content. Women showed less pronounced patterns in this regard. Nevertheless, the affective ratings of the women to the opposite- and same-sex content were also often in accordance with their self-reported sexual attraction. The EPPS does not only contain images that received a positive rating of high pleasantness, but also aversive or negatively perceived ones. This goes in line with previous research, showing that although sexual stimuli are typically processed as pleasant, they can also provoke levels of negative emotional response (Bradley et al., 2001a; Lalumière et al., 2022; Larsen & McGraw, 2011; Suschinsky & Lalumière, 2011; Staley & Prause, 2013). Thus, researchers interested in disentangling emotional and sexual responses have better access to sexual stimuli that are representative of the entire affective space.

Regarding the affective space of the EPPS images compared to the IAPS control pictures, different relationships were found between arousal and the affective ratings of valence, dominance, and disgust. As previously reported with respect to other picture sets (Bradley & Lang, 2000; Carretié et al., 2019), a significant correlation between EPPS valence and arousal ratings was found. However, we have not seen an increase in arousal as valence decreases like Carretié et al. (2019), but a positive correlation between arousal and valence ratings, which may relate to the sexual nature of the stimuli (Wierzba et al., 2015). For the sexually explicit images of the EPPS, emotional arousal increases as ratings of valence become increasingly more pleasant and disgust decreases, while feelings of control are maintained. The opposite outcome becomes apparent when analyzing the IAPS stimuli. For the assessed IAPS pictures, emotional arousal increases as ratings of valence become increasingly more unpleasant and more disgusting, and feelings of less control over the affective state. These findings to the IAPS control pictures are in line with previous research (Carretié et al., 2019; Lang & Bradley, 2007; Moltó et al., 2013). The observed differences between the assessed affective ratings (i.e., valence, arousal, dominance, disgust, moral and ethical acceptance) of the EPPS and the control IAPS images (i.e., pleasant sports scenes, neutral scenes of humans, unpleasant scenes of mutilation) further illustrate the different affective character of the sexually explicit images of the EPPS compared with the IAPS control pictures. Such observed differences between the affective space of the EPPS and the IAPS stimuli relate, on one hand, to the very sexually explicit contents of the EPPS, and on the other hand, to the inclusion of very unpleasant and highly arousing content of mutilation for the IAPS images, two differing extremes in the affective space. Pictures of erotica are known to highly activate the appetitive system, whereas threatening stimuli such as the ones selected in our study highly activate the defense system (Lang & Bradley, 2007). Additionally, some differences based on the participants’ gender and sexual attraction in the relationships between the affective ratings were observed, which need further exploration. It is not clear why, for example, no significant correlations were found between arousal and dominance to EPPS images in M-SA and W-OA, while there were medium-level positive correlations in M-OA and W-SA. Indeed, prior studies on affective ratings of arousal, valence, and dominance revealed sex differences (Bradley et al., 2001b). However, these findings were not contextualized in relation to the participants' sexual orientation. Therefore, our results may relate to gender-related traits of social roles within women and men of different sexual interests (Lippa, 2002).

The analyses presented here suggest that gender and sexual attraction play a role in affective ratings towards pornographic content. Consistent with previous research, the responses of women who were exclusively attracted to the opposite-sex were less specific than those of the other participant groups (Chivers et al., 2010; Ziogas et al., 2020). Interaction effects were found between gender, sexual attraction, and content of the images. In general, the shown content seems to be related to all affective ratings obtained (i.e., valence, arousal, dominance, and disgust and moral and ethical acceptance) towards pornographic stimuli. However, small effect sizes must be noted in this regard. Consequently, and consistent with previous research (Rupp & Wallen, 2009; Wierzba et al., 2015), the image content and the viewer's gender and sexual attraction play a role in the affective perception of pornographic material. There were preferences for certain types of images, indicating gender- and sexual attraction-specific preferences.

The sexual preferences of the observer play an important role in the affective processing of sexual stimuli (Waismann et al., 2003; Wierzba et al., 2015), which has also been observed in this study. In this regard, experimental and clinical studies using diverse sexual visual material can help to better understand human sexuality and its diversity (Stolerú et al., 2012). For example, the role of sexual media in young adulthood has already been extensively studied (e.g., Ballester-Arnal et al., 2017; Træen et al., 2006; Morgan, 2011; Ward, 2003; Wright et al., 2022). Because young adulthood is a time of sexual exploration and development (Arnett, 2014; Lefkowitz & Gillen, 2006), it is not surprising that sexually explicit media may also be an important source of information for young adults (Ballester-Arnal et al., 2017; Træen et al., 2006; Ward, 2003; Wright et al., 2022). The frequency and types of pornography use also appear to be associated with higher sexual preferences for certain types of sexual practices (Morgan, 2011). The use of pornography may play an important role in a variety of aspects of sexual development processes. In this context, researchers have discussed possible negative effects of viewing sexually explicit media, such as lower sexual and relationship satisfaction, problematic pornography consumption and increased sexual risk behaviors (Ballester-Arnal et al., 2017, 2021; Li et al., 2022; Morgan, 2011). In addition, an increase in aggressive content in pornographic videos has also been reported, with such aggressive and violent pornography repeatedly being associated with problematic outcomes such as sexually aggressive attitudes and behaviors (Bártová et al., 2021; Bridges et al., 2010, 2016; Carrotte et al., 2020; Vandenbosch & van Oosten, 2017). These data particularly point to the clinical importance of the availability of standardized materials to implement methodologically comparable experimental research in this field. Thus, material such as the EPPS can also give insights for evaluating and treating sexual disorders and relevant societal problems (e.g., sexual arousal dysfunctions, compulsive sexual behaviors such as problematic pornography consumption or paraphilic disorders). In particular, research measuring both central or peripheral correlates (e.g., fMRI, MEG, EEG, facial EMG, autonomic activity) to enhance the understanding of neurobiological underpinnings of human sexuality and sexual disorders, may benefit from a stimuli set with a range of different sexually explicit pictures. To this extent, studies in the field have so far lacked inclusion of diverse populations since predominantly heterosexual male participants have been studied (van’t Hof & Cera, 2021).

To summarize, EPPS images vary in terms of affective ratings and provide nuanced pleasant/unpleasant, arousing, and varying degrees of disgusting, and ethically and morally acceptable sexual scenes to deepen our understanding of human sexuality by facilitating research in this area, also from a clinical point of view. In addition, physical image parameters that have been shown to be important in modulating the affective impact of visual stimuli were calculated and analyzed (Carretié et al., 2019; Knebel et al., 2008; Lakens et al., 2013). Our analyses further highlighted significant associations indexing physical–affective relationships. Arousal and valence have been shown to correlate with the color space and complexity of the EPPS images. The given affective ratings in combination with the provided physical image parameters for each image should be able to guide the selection of stimuli according to the requirements of different experimental designs.

Nevertheless, there are some limitations to this study. First, the EPPS ratings were collected from a population of rather young adults with a high educational background, recruited mainly at universities. Given that there is some evidence for effects of age and sociocultural factors on valence and arousal ratings and emotion processing (Gruhn & Scheibe, 2008), a replication study in an older sample and from other sociocultural backgrounds should be a future goal. Even though Spanish samples seem to be essentially similar in respect to the assessments of emotional pictures (Moltó et al., 1999; Moltó et al., 2013; Soares et al., 2015; Vila et al., 2001), cultural differences in the evaluation of the affective scenes are possible and should be further assessed. Secondly, the EPPS contains mainly pleasant and highly arousing images. Consequently, this new picture set is asymmetric in this sense, lacking rather neutral images. According to the experimental literature, neutral stimuli tend to be assessed from low to medium emotional arousal, and score in the mid-range of affective valence ratings (Ruiz-Padial et al., 2021). As sexual stimuli rarely fall into this category, a sample of IAPS control pictures (Lang et al., 1999) were accordingly included in the standardization process of the EPPS. Nevertheless, filling a research gap, the EPPS offers a range of pleasant/unpleasant and arousing, as well as more disgusting and less ethically and morally acceptable, sexually explicit images. Apart from subjective ratings indicating differences related to viewers’ gender and sexual attraction, such differences in EPPS ratings should be further validated by measuring central and peripheral psychophysiological correlates (e.g., fMRI, EEG, electrodermal and cardiovascular activity, facial EMG, startle reflex, eye-tracking, or penile and vaginal photoplethysmography). Further monitoring of hormone levels would also be recommended, as hormonal fluctuations due to menstrual cycle or contraceptive use may influence the perception of sexual stimuli (Renfro et al., 2015; Shirazi et al., 2018; Suschinsky et al., 2014; Wallen & Rupp, 2010). It is unclear how far hormonal fluctuations in males may influence such processes (Hardy et al., 2005; Wu et al., 2022). Therefore, future studies should investigate any of the above physiological biomarkers of individuals in relation to their affective perception towards different sexual stimuli, including a more diverse sampling in terms of gender identities and a more nuanced assessment of sexual interests.

Despite the above limitations and methodological improvements that could be implemented over time, the EPPS adds to the current literature as it constitutes a reliable pictorial set comprising a wide range of pornographic images normed on both dimensional and discrete affective ratings. In addition to providing important physical picture parameters, it opens new avenues for basic and clinical experimental designs in emotion and sexuality research.

## Conclusions

The EPPS dataset offers standardized explicit pornographic pictures of diverse sexual practices (masturbation, oral sex, vaginal sex, anal sex, group sex, paraphilia) of opposite- and same-sex pornography. Normative ratings of valence, arousal, and dominance, as well as disgust and moral and ethical acceptance ratings, were available of participants with sexual attraction to either exclusively the opposite-sex or also the same-sex. Additionally, several physical characteristics were computed to provide relevant picture properties (width, height, luminance, contrast, complexity, entropy, and color composition), allowing for an easier and more accurate selection of physically matching stimuli. The EPPS dataset is freely available for the research community on OSF (https://osf.io/s5dhb/?view_only=9f54594ec51641a484da36ef0bf1c4e6), upon request to the corresponding author, in order to enhance a certain level of comparability across studies using visual sexual stimuli, and to facilitate affective picture selection for experimental and clinical research.

## Data Availability

Data to instructions, as well as the affective ratings and physical properties of each image, are available as online supplementary material on OSF (https://osf.io/s5dhb/?view_only=9f54594ec51641a484da36ef0bf1c4e6). The images of the EPPS can be acquired in color or gray format for noncommercial research purposes by contacting the corresponding author through email (mpastor@uji.es). This approach had to be chosen in order to fulfill the collaboration agreements with the pornography producers and distributors.

## Footnotes

1 The included IAPS stimuli are the following: 3400 (mutilated arm), 3130 (mutilated upper body), 3120 (mutilated upper body), 3110 (burned upper body), 3060 (mutilated face), 3030 (mutilated face), 3010 (mutilated face), 3071 (mutilated neck), 8341(aerobatics plane), 8193 (man skiing), 8178 (ski diver on a waterfall), 8191(man doing ice climbing), 8186 (man doing sky snowboarding), 8185 (group of skydivers), 8179 (boy bungee jumping), 5621 (group of skydivers), 2745.1 (female shopping), 2575 (mechanic working), 2396 (couple coming downstairs), 2104 (female looking into camera), 2102 (man reading the newspaper), 2036 (female shopping), 2026 (cashier), 2513 (elder female looking into camera).
